# Nifuroxazide boosts the anticancer efficacy of palbociclib-induced senescence by dual inhibition of STAT3 and CDK2 in triple-negative breast cancer

**DOI:** 10.1038/s41420-023-01658-w

**Published:** 2023-09-26

**Authors:** Xianzhe Wang, Wei Shi, Xumei Wang, Jin-Jian Lu, Ping He, Hongjie Zhang, Xiuping Chen

**Affiliations:** 1 State Key Laboratory of Quality Research in Chinese Medicine, Institute of Chinese Medical Sciences, University of Macau, Macao, China; 2 Department of Pharmacology, School of Pharmacy, Guangxi Medical University, Nanning, Guangxi China; 3 Biological Imaging and Stem Cell Core, Faculty of Health Sciences, University of Macau, Taipa, Macao China; 4 MoE Frontiers Science Center for Precision Oncology, University of Macau, Taipa, Macao China; 5 GMU-GIBH Joint School of Life Sciences, The Guangdong-Hong Kong-Macau Joint Laboratory for Cell Fate Regulation and Diseases, Guangzhou Medical University, Guangzhou, China

**Keywords:** Drug development, Breast cancer

## Abstract

Though palbociclib, a cyclin-dependent kinases 4 and 6 (CDK4/6) inhibitor has been approved for treating breast cancer, two major clinical challenges remain: (i) Triple-negative breast cancer (TNBC) appears to be more resistant to palbociclib, and (ii) Palbociclib-induced senescence-associated secretory phenotype (SASP) has a pro-tumorigenic function. Here we report that combining palbociclib with the STAT3 inhibitor nifuroxazide uncouples SASP production from senescence-associated cell cycle exit. Moreover, we identified nifuroxazide as a CDK2 inhibitor that synergistically promotes palbociclib-induced growth arrest and senescence in TNBC cells. In vitro, the combination of nifuroxazide with palbociclib further inhibited the TNBC cell proliferation and enhanced palbociclib-induced cell cycle arrest and senescence. The modulation of palbociclib-induced SASP by nifuroxazide was associated with the reduction of phosphorylated-STAT3. Nifuroxazide also blocks SASP-dependent cancer cell migration. Furthermore, thermal shift assay and molecular docking of nifuroxazide with STAT3 and CDK2 revealed that it binds to their active sites and acts as a potent dual inhibitor. In vivo, the combination of nifuroxazide with palbociclib suppressed 4T1 tumor growth and lung metastasis. Our data suggest that nifuroxazide enhances the anticancer effects of palbociclib in TNBC by uncoupling SASP production from senescence-associated cell cycle exit and inhibiting CDK2 to promote tumor senescence.

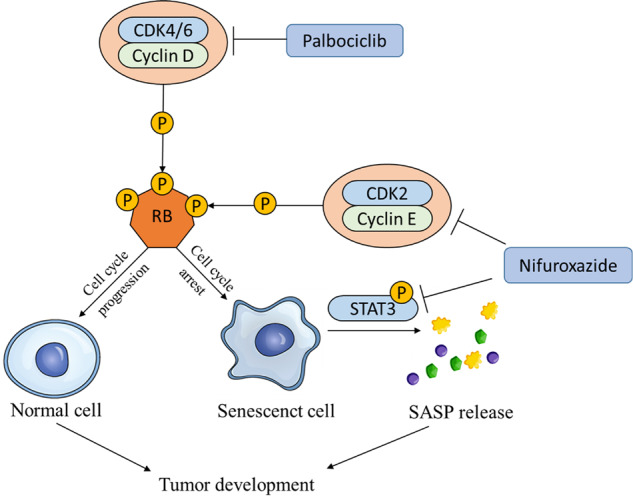

## Introduction

Breast cancer is the most common type of cancer among women worldwide, and continues to be one of the common causes of cancer-related deaths with about 2.3 million new cases annually [1]. The clinical treatment for breast cancer mainly depends on the molecular phenotype of the tumor [2]. Triple-negative breast cancer (TNBC), which lacks estrogen receptor (ER), progesterone receptor (PR), and human epidermal growth factor receptor 2 (HER2), is a highly aggressive subtype of breast cancer. Although it accounts for approximately 15–20% of all breast cancers [3], it contributes significantly to tumorigenesis and resistance to chemotherapy and is thus associated with an increased risk of breast cancer recurrence and death [4]. Currently, there are few therapeutic options to control the recurrence and metastasis of TNBC. Therefore, new therapeutic strategies for TNBC treatment are urgently needed.

Palbociclib (Ibrance, Pal) is a selective inhibitor of cyclin-dependent kinase 4/6 (CDK4/6) approved by the Food and Drug Administration (FDA) in 2015 for the treatment of hormone receptor-positive (HR^+^)/HER2 negative (HER2^−^) breast cancer in combination with hormone therapies [5]. The anticancer effect of Pal is mainly due to its ability to disrupt the cell cycle process and induce cancer cell senescence [6, 7]. Promising clinical advances have encouraged Pal treatment in TNBC patients [8]. However, TNBC is less sensitive to CDK4/6 inhibition through poorly understood mechanisms. The activation of the cyclin E–CDK2 axis in TNBC is one possible mechanism that confers resistance to Pal [9]. Another possible mechanism is the induction of senescence-associated secretory phenotype (SASP) by Pal, which creates an inflammatory and immunosuppressive microenvironment that promotes tumor progression, metastasis, and resistance to cancer therapies [10]. Thus, targeting CDK2 and modulating SASP may enhance the anticancer efficacy of CDK4/6 inhibitors in TNBC.

Janus kinase/signal transducer and activator of transcription (JAK-STAT) pathway regulates multiple biological functions in the initiation of malignant transformation and plays a key role in regulating SASP production [11, 12]. STAT3 is the leading member of the STAT family that mediates both the release of pro-carcinogenic SASP and the suppression of anti-tumor immunity [13]. In addition, the STAT3 pathway also associates with different features of oncogenesis in breast cancer, including metastasis, higher stages of disease progression, and Pal resistance [14, 15].

Nifuroxazide (Ambatrol, Nif), a STAT3 inhibitor, was originally approved as an antidiarrheal agent [16]. Recently, it has been found to exert potent anti-proliferation, and anti-metastasis effects on breast cancer and promote the immune response against tumors [17, 18]. In this study, we found that Nif is a dual inhibitor of STAT3 and CDK2, and works synergistically with Pal to combat TNBC.

## Results

### The combination of Nif and Pal synergistically inhibited TNBC cell proliferation and enhanced senescence

**Fig. 1:**
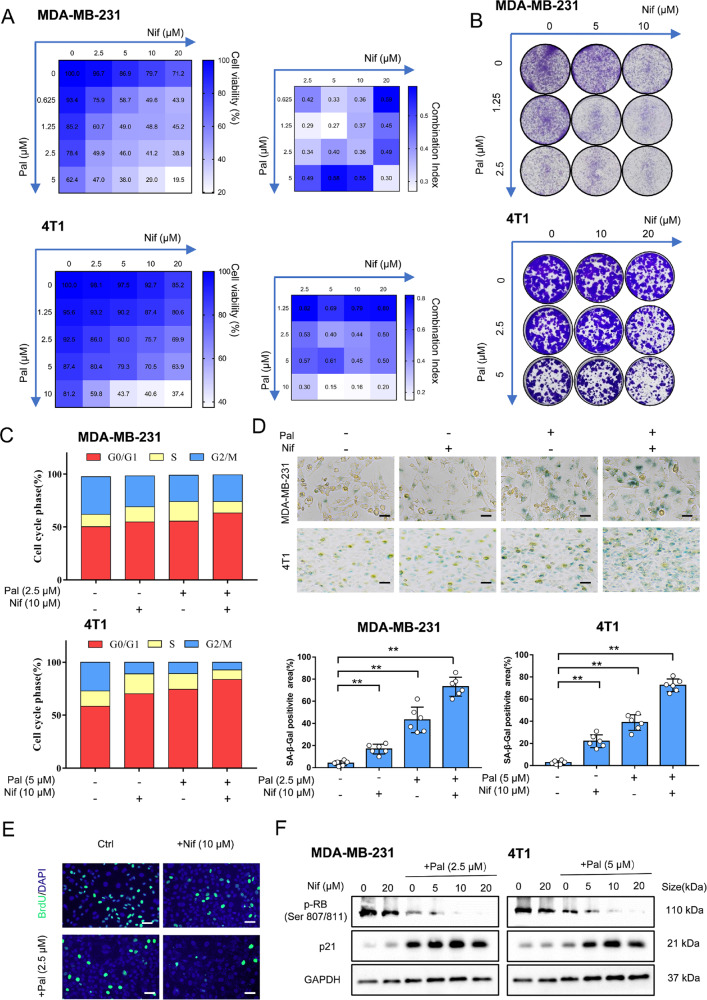
Nif synergizes with Pal to promote tumor senescence. **A** Cell viability of MDA-MB-231 and 4T1 cells after co-treated with increasing concentrations of Nif (0-20 μM) and Pal (0–10 μM) for 3 days. CI < 1 indicated a synergism effect. **B** Colony formation assays to verify the long-term anti-proliferation effect after the combination treatment for 7 days. **C** Cells were treated for 24 h, and the cell cycle distribution was detected by flow cytometry. **D** Representative images and quantification of SA-β-galactosidase staining were measured after 3 days of treatment. Green indicates positive staining. Scale Bar:25 μm. **E** Representative images of BrdU staining after 3 days of treatment. Scale Bar:50 μm. **F** Western blot analysis of p21- RB senescence signaling pathway after 3 days of treatment. All data represent mean ± S.D. from at least three independent experiments; **P < 0.01.

Pal and Nif significantly reduce cell viability in a concentration-dependent manner. The IC_50_ values of Pal after 72 h treatment were 8.7 μM and 16.7 μM for MDA-MB-231 and 4T1 cells, respectively. The IC_50_ values of Nif were 50.5 μM and 62.8 μM respectively. (Supplementary Fig. 1). The combination of Nif and Pal for 72 h synergistically inhibited cell viability (CI < 1). (Fig. 1A). Furthermore, the combination significantly inhibited the size and number of colonies formed in TNBC cell lines suggesting a long-term anti-proliferative effect (Fig. 1B). Pal treatment for 24 h induced the cell cycle arrested in the G0/G1 phase, accompanied by a decrease in the fraction of cells in the G2/M phases. The combined treatment resulted in a more efficient arrest in the G0/G1 phase and a decrease in the proportion of cells in the S and G2/M phases (Fig. 1C). Long-term cell cycle arrest is sufficient to trigger cellular senescence [19]. Pal and Nif alone slightly increased the SA-β-gal-positive cells. The combination significantly increased the proportions of SA‐β‐gal‐positive cells (Fig. 1D) and BrdU‐negative cells (Fig. 1E). In addition, for the senescence pathway, the combination of Nif and Pal decreased protein expression of p-RB and increased protein expression of p21 in TNBC cells (Fig. 1F). These results suggest that the combination of Nif further inhibit TNBC cell proliferation by enhancing senescence.

### Nif synergizes with Pal to induce senescence by targeting CDK2

**Fig. 2:**
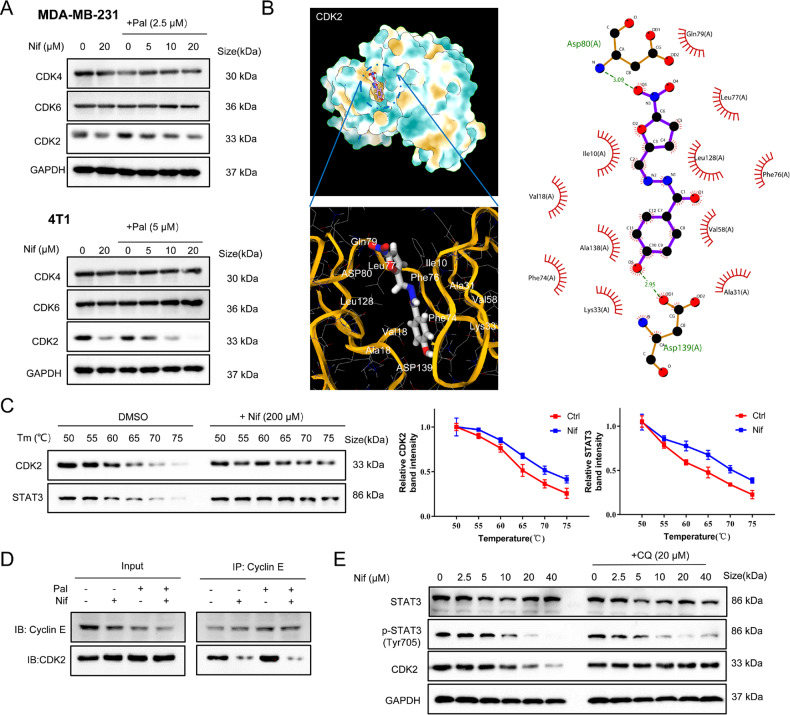
Nif interacts with both STAT3 and CDK2. **A** Western blot analysis of CDK2, CDK4, and CDK6 protein from G1 cell cycle arrest pathway after 3 days of treatment. **B** The binding mode of Nif to CDK2. 3D structures showed the binding pockets, coloring from blue (for strong hydrophobic regions) to yellow (for poor hydrophobic regions). 2D-interaction pose of Nif illustrating hydrogen bonds. **C** Thermal shift assay showing the effects of Nif on thermal stabilization of CDK2 and STAT3 protein in MDA-MB-231 cell lysates. **D** The complex formation of cyclin E and CDK2 was measured by the co-immunoprecipitation assay after 24 h of treatment. **E** Western blot analysis of STAT3, p-STAT3(Tyr705), and CDK2. MDA-MB-231 cells were pretreated with CQ (20 μM) for 2 h, together with Nif treatment for 72 h.

Given that the combination promotes cell cycle arrest in G0/G1 phase, we hypothesize that Nif enhances senescence in TNBC by controlling the cell cycle. The expressions of cyclin-dependent kinases(CDKs) involved in G0/G1 phase regulation [20, 21] were examined. Western blotting revealed that Nif dose-dependently inhibited CDK2 expression after 72 h treatment with an IC20 dose of Pal, while CDK4 and CDK6 expression remained unchanged (Fig. 2A). We then performed molecular docking to assess whether Nif could bind to CDK2 (PDB code: 1DI8). The docking results (Fig. 2B) showed that Nif interacted with the ATP-binding pocket of CDK2 through two hydrogen bonds with Asp80 and Asp139 [22]. We confirmed the in vitro binding between Nif and CDK2 protein using CETSA with whole protein lysate from MDA-MB-231 cells [23]. Nif protected the CDK2 protein from denaturation and precipitation at elevated temperatures, similar to STAT3 proteins, suggesting a dual inhibitory effect of Nif on both STAT3 and CDK2 (Fig. 2C). Since the CDK2/cyclin E complex mediates the G1/S phase transition, we wondered whether the binding of Nif would disrupt the complex formation. We performed co-IP experiments and found that Pal slightly enhanced the CDK2/cyclin E1 interaction after 24 h treatment, while Nif significantly inhibited it (Fig. 2D). In addition, disrupting their interactions could promote the autophagic degradation of CDK2 [24, 25]. From this, we treated MDA-MB-231 cells for 72 h with Nif alone or with the autophagy inhibitor CQ. Autophagy inhibition significantly blocks Nif-induced CDK2 degradation. These results suggest that the STAT3 inhibitor Nif may bind and inhibit CDK2 and impair the CDK2/cyclin E complex formation.

### Nif suppresses Pal-induced SASP release by inhibiting STAT3 phosphorylation

**Fig. 3:**
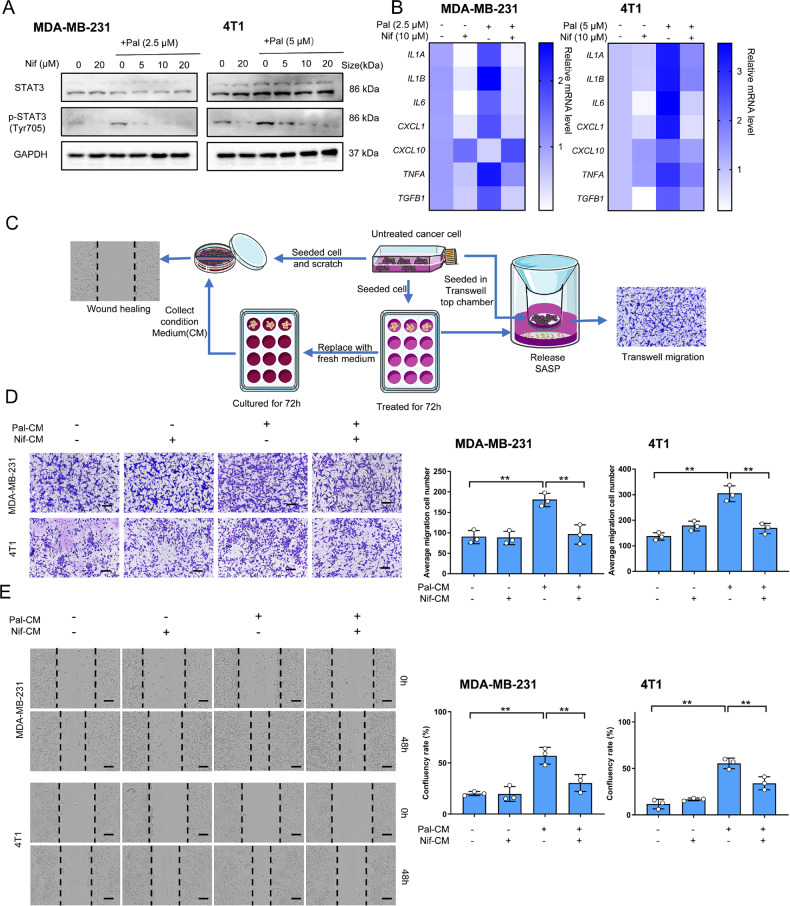
Nif modulated the profiles of Pal-induced SASP by inhibiting STAT3. **A** Western blot analysis of STAT3 and p-STAT3(Tyr705) protein after 3 days of treatment. **B** Gene expression analysis of SASP components by real-time qPCR after 3 days of treatment. Results (fold change vs. ctrl) are presented from three independent experiments. **C** Schematic of co-culture experiments to assess the impact of SASPs from 3-day treatments. Normal cells are co-cultured with indicated treated cells or exposed to conditioned medium. **D** Representative images and quantification of transwell migration assays of cells co-cultured with the indicated treated cells for 24 h. Scale bar:200 μm. **E** Representative images of scratched cells cultured in conditioned medium from indicated treated cells for 48 h. Scale bar:200 μm. All data represent mean ± S.D. from at least three independent experiments; **P < 0.01.

STAT3 pathway in senescent tumors establishes a SASP-dependent microenvironment that contributes to tumor progression and therapy resistance [14]. STAT3 inhibition can uncouple SASP production from senescence-associated cell cycle exit [19]. To determine the role of Nif as a STAT3 inhibitor in combination with Pal, we assessed the status of STAT3 phosphorylation in TNBC cells. Nif co-treatment significantly inhibited Pal-induced STAT3 phosphorylation (Tyr705) (Fig. 3A). qPCR confirmed Nif decreased SASP release (*IL1A, IL1B, IL6, CXCL1, TNFA*, and *TGFB1*) in Pal-induced senescent cells (Fig. 3B). One of the main impacts of SASP is the remodel of the tumor microenvironment that promotes cancer cell metastasis [11]. Therefore, we used a transwell co-culture system to ensure that senescent cells and untreated cancer cells were physically separated while sharing condition medium (CM) with secreted soluble SASP factors (Fig. 3C). Pal-induced senescent cells secreting SASP significantly promoted the migration of untreated cancer cells after 24 h co-culture. Co-culture with cells from the Nif co-treatment group inhibited the migration of MDA-MB-231 and 4T1 cells (Fig. 3D). The wound healing assay obtained similar results (Fig. 3E). SASP and STAT3 pathways were capable of promoting resistance to chemotherapy-induced apoptosis [13]. The combination with Nif increased the sensitivity of Pal-treated cells to cisplatin-induced apoptosis (Supplementary Fig. 2). These results suggest that combined with Nif suppresses Pal-induced SASP release by inhibiting STAT3 phosphorylation and further abolished the SASP-mediated effects in surrounding microenvironment.

### CDK2 and STAT3 are complementary targets in enhancing the anticancer effects of Pal

**Fig. 4:**
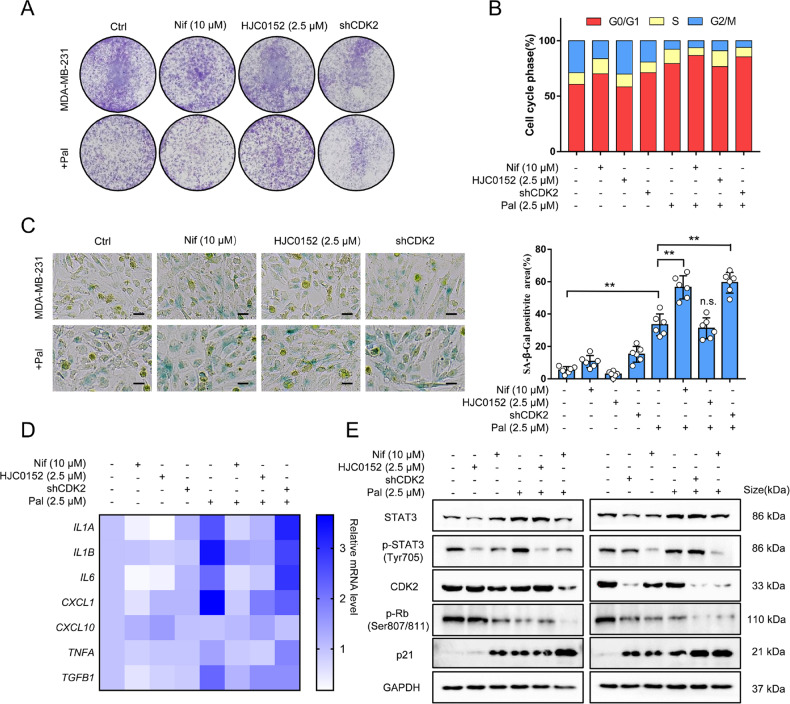
CDK2 and STAT3 are independent targets of Nif. **A** Colony formation assays to compare the anti-proliferation effect of the combined treatment with STAT3 inhibitor HJC0152, transfection with CDK2 shRNA or Nif on MDA-MB-231 cells. **B** Cells were treated for 24 h, and the cell cycle distribution was detected by flow cytometry. **C** Representative images and quantification of SA-β-galactosidase staining were measured after 3 days of treatment. Green indicates positive staining. Scale Bar:25 μm. **D** Gene expression analysis of SASP components by real-time qPCR after 3 days of treatment. Results (fold change vs. ctrl) are presented from three independent experiments. **E** Western blot analysis of STAT3, p-STAT3(Tyr705), CDK2, pRB(Ser807/811), and p21 protein expression after 3 days of treatment. All data represent mean ± S.D. from at least three independent experiments; **P < 0.01.

To investigate whether Nif inhibits STAT3 and CDK2 independently or through overlapping pathways, we determined the combination effect of HJC0152 (another specific STAT3 phosphorylation inhibitor) and shCDK2 (a CDK2 knockdown vector) with Pal. Nif or shCDK2 but not HJC0152 synergistically reduced the colony formation (Fig. 4A), increased G1 phase arrest (Fig. 4B), and SA-β-gal-positive cells (Fig. 4C) when combined with Pal, suggesting CDK2 rather than STAT3 involved in Pal-induced proliferation inhibition.

However, a combination with HJC0152 or Nif but not shCDK2 could inhibit the SASP release (Fig. 4D), indicating the regulatory role of STAT3 in SASP release. Moreover, Nif inhibited both STAT3 phosphorylation and CDK2 expression, while HJC0152 or shCDK2 failed to do so (Fig. 4E). Thus, Nif, as a dual inhibitor, has the potential to complement CDK2 and STAT3 in enhancing the anticancer efficiency of Pal.

### Combining Nif with Pal suppresses tumor proliferation in vivo

**Fig. 5:**
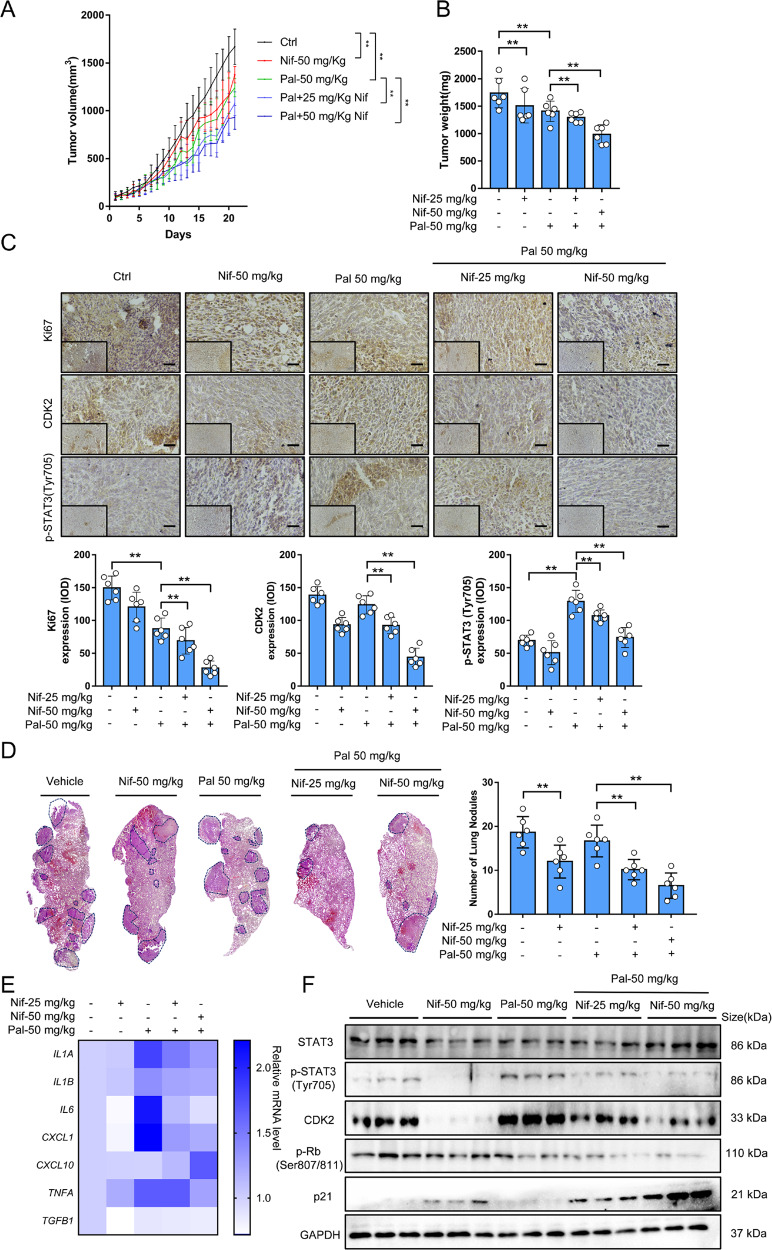
Nifuroxazide synergizes with Palbociclib to inhibit tumor growth in TNBC xenograft mice. **A** 4T1 xenograft tumor growth curve relative to the treatment starting point (week 8, normalized to Day 0) upon separate or combined treatment with vehicle, Nif (25 mg/kg/d or 50 mg/kg/d, i.p.), and Pal (50 mg/kg/d, i.g.), (*n* = 6 tumors per group) daily for 21 days. **B** Tumor weight at the endpoint of treatment (week 11). **C** Representative images and quantification of immunohistochemistry results (400×) of Ki67, CDK2, and p-STAT3(Tyr705) in tumors. Scale Bar:100 μm. **D** Representative images and quantification of H&E staining results of metastasis nodules in the lungs of mice bearing 4T1. The blue frame indicates tumor metastasis nodules. **E** Gene expression analysis of SASP components by real-time qPCR of 4T1 xenograft tumors. **F** Western blot analysis of STAT3, p-STAT3(Tyr705), CDK2, pRB(Ser807/811) and p21 protein expression of 4T1 xenograft tumors. All data represent mean ± S.D. from at least three independent experiments; **P < 0.01.

To illustrate the synergistic effect, low dosages of Pal (50 mg/kg/d) and Nif (25 and 50 mg/kg/d) were selected [17, 26]. Treatments did not affect body weight or induce liver or kidney pathological changes (data not shown). Both Nif and Pal alone yield slight therapeutic responses while the combination significantly inhibited tumor growth (Fig. 5A) and decreased tumor weight (Fig. 5B). IHC results showed that the combination decreased the expression of Ki67, CDK2, and p-STAT3 expression in tumor tissues, while Pal alone showed elevated CDK2 expression and upregulated p-STAT3 expression (Fig. 5C). The 4T1 tumor has a strong metastatic potential and spontaneously metastasize to lung as early as two weeks after inoculation [17]. Furthermore, we observed multiple metastases in the lungs of the vehicle and Pal groups, which were significantly decreased in Nif and combination groups (Fig. 5D). The combination decreased the SASP levels in tumors (Fig. 5E). Nif combination treated xenograft tumors showed decreased expression of p-STAT3, p-RB, and CDK2, and increased expression of p21 (Fig. 5E). To offer a glimpse into future clinical applications, we analyzed the gene expression of Pal targeted genes, CDK4 and CDK6, and Nif targeted genes STAT3 and CDK2 in breast cancer through the TNMplot database [27]. The gene signatures of the Pal and Nif targeted genes have a higher score in breast tumor and metastasis breast tumors, which reveals therapeutic intervention on co-targeting these genes was likely to benefit clinical treatment (Supplementary Fig. 3).

## Discussion

CDK4/6 inhibitors show promising anticancer activity in ER-positive breast cancer [14]. However, their efficiency was compromised by the insensitivity and the pro-tumorigenic function of SASP in TNBC [9]. Here, we identify that Nif is able to inhibit CDK2, which synergistically promotes cell cycle arrest and senescence with Pal in TNBC. In addition, Nif suppresses Pal-induced pro-tumorigenic SASP release as well as SASP-dependent microenvironment.

Our study used two TNBC cell lines, MDA-MB-231 human breast cancer cells, and 4T1 murine mammary carcinoma cells. In line with previous studies [8, 28], 4T1 cells were less sensitive to Pal treatment, while Nif treatment showed no significant difference between the two cell lines [17]. CDK4/CKD6 expression levels and p21-pRB signaling pathways levels showed no significant differences between the two cells. 4T1 cells, however, had relatively higher levels of p-STAT3 and CDK2 expression than MDA-MB-231 cells, and Pal treatment also caused a higher level of SASP release which was consistent with the fact that 4T1 cells are a rich source of inflammatory mediators [29], which may result in the insensitivity of 4T1 cells to Pal treatment.

The cyclin E-CDK2 signaling has been highlighted as a bypass mechanism to escape CDK4/6 inhibition by Pal [30–32]. Both CDK2 and CDK4/6 are kinases that regulate the G1/S phase cell cycle transition. Previous studies showed that combining CDK2 and CDK4/6 inhibition could overcome Pal-resistant in ER-positive breast cancer [32, 33]. And Pal-resistant TNBC cells exhibited higher levels of CDK2 [34]. Here, we observe Pal-induced G0/G1 phase cell cycle arrest, which is enhanced by Nif by inhibiting CDK2-promoting senescence. We demonstrate that Nif is able to interact with the CDK2 protein, impairing the formation of the CDK2-Cyclin E1 complex and, consequently, facilitating the autophagic degradation of the non-functional CDK2. Although the CDK2 knockdown mimics the boost effect of Nif on proliferation inhibition and senescence, it does not affect SASP release as much as Nif does.

CDK4/6 inhibitors eliciting SASPs is a common effect [7]. SASP showed dual effects on cancer progression: some components stimulated tumor growth, survival, and metastasis, while others reinforced senescence and promoted immune clearance [28, 35]. We found that Pal-treated TNBC cells produced a SASP-release phenotype with an increased pro-tumorigenic SASP component. Emerging evidence suggested these components were modulated by STAT3 signaling [36]. Our data reveal that STAT3 is robustly phosphorylated by Pal and that Nif inhibits STAT3 phosphorylation and decreases the mRNA levels of Pal-induced SASP. This finding was consistent with the observation that STAT3 inhibitors reprogrammed the SASP in senescent tumor cells, leading to improved antitumor response [11]. HJC0152, a specific STAT3 inhibitor, also inhibits SASP release and abolishes the SASP-mediated effects in the surrounding microenvironment. However, HJC0152 did not show any effect in promoting Pal-induced proliferation inhibition. These results suggest that Nif, as a dual inhibitor of CDK2 and STAT3, may complement each other in enhancing the antitumor effects of Pal.

In conclusion, our study reveals that Nif is a dual inhibitor of CDK2 and STAT3. Nif in combination with Pal has been shown to have a synergistic effect on TNBC by promoting growth arrest and senescence and inhibiting the release of SASP induced by Pal. Our findings provide a novel therapeutic combination for TNBC.

## Materials and methods

### Chemicals and reagents

Nif and Pal were purchased from Shanghai Aladdin Biochemical Technology (Shanghai, China). Chloroquine and HJC0152 were obtained from Selleck Chemicals (Houston, TX, USA). Antibodies against p-Rb (Ser807/811) (#9308), cyclin E1 (#20808), p-STAT3 (Tyr705, #9145), STAT3 (#9139), CDK4 (#2906), CDK6 (#3136), CDK2 (#2546) and GAPDH (#2118) were purchased from Cell Signaling Technology (Beverly, MA, USA). Antibodies against p21 (AF5252) were purchased from Beyotime Biotechnology (Shanghai, China).

### Cell culture

The human breast cancer cell line MDA-MB-231 and the mouse mammary carcinoma cell line 4T1 were obtained from the American Type Culture Collection (ATCC, Rockville, MD, USA), Cells were cultured in Dulbecco modified Eagle medium (DMEM) and RPMI 1640, respectively, supplemented with 10% fetal bovine serum (FBS) and 1% penicillin/streptomycin at 37 °C in a 5% CO_2_ atmosphere.

### Cell viability

Cells seeded (4 × 10^3^/well) in 96-well plates were allowed to adhere overnight and then treated with Nif (0–20 μM for MDA-MB-231 and 4T1 cells) and Pal (0–5 μM for MDA-MB-231 and 0–10 μM for 4T1 cells) for 3 days. Cell viability was assessed by MTT assay according to the manufacturer’s instructions. Synergy analysis was performed using the Compusyn software by Chou–Talalay method [37]. The synergy analysis was determined based on the combination index (CI): synergism (CI < 1), additive effect (CI = 1), or antagonism (CI > 1).

### Cell proliferation

For the colony formation assay, cells were seeded in specified numbers (100 cells/well) in 12-well plates to adhere overnight. Then the cells were co-treated with Nif (0–20 μM) and Pal (0–5 μM) for additional 7 days. The cells were fixed and stained with a 0.5% crystal violet solution for 15 min and the colonies were observed under a microscope. For bromodeoxyuridine (BrdU) analysis, cells in 96-well after indicated treatment for 3 days were pulsed with 10 µM BrdU (Beyotime, Shanghai, China) for 5 h, trypsinized, fixed, and labeled according to the manufacturer’s instructions.

### Cell cycle analysis

Cells were harvested after 24 h indicated treatments and then washed with PBS, fixed in pre-chilled 70% ethanol, and kept overnight at 4 °C. The fixed cells were then collected, washed, and resuspended in PBS. The cells were incubated with 1 mg/mL RNase and 50 µg/mL propidium iodide (PI) in the dark for 30 min at room temperature and subjected to flow cytometry (BD Biosciences, CA, USA). The cell cycle results were analyzed using FlowJo version 7.6.1.

### Senescence-associated β-galactosidase (SA-β-gal) staining

Senescence was measured by the senescence-associated β-galactosidase staining kit (Beyotime, Shanghai, China) according to the manufacturer’s instructions. Briefly, cells (6 × 10^4^/well) were plated in 24-well plates and treated for 3 days. Cells were then stained with SA-ß gal solution overnight and observed under the microscope. The senescent cells were quantified by counting 100 cells in three different fields for each replicate.

### shRNA knockdown

Stable CDK2-shRNA knockdown cells were generated as described previously [38]. CDK2 shRNA (5‘-CTCCTGGGCTGCAAATATTAT-3‘) was constructed by Beyotime Biotechnology (Shanghai, China). To generate lentivirus-expressing shRNA, HEK293T cells were transfected with pLKO-Tet-Puro vector (scrambled shRNA or shRNA against CDK2) according to the manufacturer’s protocol. After 48 h of transfection, the virus-containing medium was collected, filtered, and added to the cells of MDA-MB-231 in the presence of 8 μg/mL of polybrene (Millipore). The lentivirus-infected cells were selected with 1 μg/mL puromycin (InvivoGen, San Diego, CA), and verified in this study by western blotting.

### Molecular docking

The CDK2 crystal structures were obtained from RCSB Protein Data Bank (PDB ID: 1DI8) [39]. The molecules of Nif were obtained from PubChem and minimized the energy by Chem3D. The CDK2 and Nif were docked by SwissDock (http://www.swissdock.ch/) [22]. The docking results were visualized by Chimera 1.13.1 and Ligplot 2.0.

### Cellular thermal shift assay

The cellular thermal shift assay was performed according to the literature described [23]. Cells were cultured as mentioned above for protein extraction. The protein lysate was divided into 6 equal parts and heated at 50, 55, 60, 65, 70, and 75 °C for 3 min. After centrifuging the samples at 15,000 rpm for 15 min, the final protein was obtained by removing the supernatant. Then, protein images were obtained and analyzed according to the western blotting.

### Quantitative real-time PCR

The total RNA of cells and tissues was extracted by RNA Isolation Kit with Spin Column (Beyotime, Shanghai, China) and cDNA was synthesized using the Transcriptor First Strand cDNA Synthesis Kit (Beyotime, Shanghai, China). qRT-PCR was performed with SYBR Green I Master Mix (Roche) on ViiA 7 Real-Time PCR System (Thermo Fisher, MA, US). The PCR primers are listed in Supplementary Table 1. All results were normalized to those for GAPDH.

### Transwell assay

MDA-MB-231 cells were seeded (4 × 10^3^/well) in Transwell insert top chamber. In parallel, MDA-MB-231 cells were seeded and treated on 12-well plates for 3 days, and then resuspended and seeded (5 × 10^4^/well) in the bottom chamber. After 24 h culture, cells on top of each insert were removed and the transwell membrane was mounted onto slides. The migration of cells to the bottom of the membrane was stained with a 0.5% crystal violet solution for 15 min and visualized under a microscope.

### Wound healing assay

Cells were seeded (6 × 10^5^/well) in 6-well plates. The cell monolayers to a confluence of about 80% in 6-well plates were scraped by a p200 pipette tip, and conditional medium from indicated treated groups after centrifugation at 3000 rpm for 10 min was added. After 48 h incubation, images were acquired using Incucyte (Essen Bioscience, Hertfordshire, UK), and the confluency rate of migrated cells was compared to the value assigned for the ctrl medium treated group.

### Animal studies

4T1 cells (5 × 10^5^) were injected into the mammary gland 3 fat pad armpit of 8 weeks female Balb/c mice. To illustrate the synergistic effect, relatively low dosages of Pal (50 mg/kg/d) and Nif (25 mg/kg/d and 50 mg/kg/d) were selected [17, 26]. When the tumors reached a mean size of 100 mm^3^, the mice were randomized into 5 groups: vehicle, Nif alone or combination (25 mg/kg/d or 50 mg/kg/d), and Pal alone or combination (50 mg/kg/d). Each group has 6 mice (*n* = 6). Pal was dissolved in 0.5% CMC-Na and was orally administered daily. Nif was dissolved in dissolvent (5:25:70 ratio of DMSO: HS-15: normal saline) and administered by intraperitoneal injection. Body weights and tumor sizes were recorded every day. Mice were sacrificed 3 weeks after treatment. The tumors and lung tissues were collected and weighed. Tumor volume (V) was estimated according to the formula: V = (length × width^2^)/2. All the animal procedures were conducted under the Guidelines for the Care and Use of Laboratory Animals and were approved by the Animal Research Ethics Committee at the University of Macau (UMARE-014-2021).

### H&E staining and immunohistochemistry

H&E staining was performed using a commercial kit (Solarbio, Beijing, China). IHC staining was performed on paraffin-embedded tumor tissue sections (6 μm) using an IHC staining kit (ZSGB-BIO, Beijing, China) as previously described [6]. Sections were stained with primary antibodies and followed using a DAB Detection Kit (ZSGB-BIO, Beijing, China). Staining was visualized under a microscope and quantified by Image J software.

### Western blotting

The western blotting was performed as described previously [6]. Cells were seeded in (3 × 10^5^/well) in 6-well plates and lysed in RIPA buffer. For mouse tissues, the tumors were minced into small pieces on dry ice and immersed in RIPA buffer for lysis. A total of 30 μg protein per sample was separated by SDS-PAGE gel electrophoresis and transferred onto PVDF membranes. The blot signal was visualized using the ChemiDoc™ MP Imaging System.

### Statistical analysis

Statistical analysis was performed with GraphPad Prism 5.0 (GraphPad Software, San Diego, CA, USA) and SPSS 23.0 software (IBM, Armonk, NY, USA). All data represent at least 3 independent experiments and are expressed as mean ± S.D. Statistical comparisons from multiple groups were made using one-way ANOVA with the Tukey procedure to adjust for multiple comparisons. *P*-values of less than 0.05 were considered to represent statistical significance.

### Supplementary information

Supplementary Material

Original Data File

## Data Availability

Data will be made available on request.
