# Supplementary material for: Nifuroxazide boosts the anticancer efficacy of palbociclib-induced senescence by dual inhibition of STAT3 and CDK2 in triple-negative breast cancer
Source: Cell Death Discov. 2023 Sep 26;9:355. doi: 10.1038/s41420-023-01658-w (PMC10522654; doi:10.1038/s41420-023-01658-w)
Supplement: Supplementary file 1 — Supplementary Material [file 41420_2023_1658_MOESM1_ESM.docx]

SUPPLEMENTARY TABLE

Supplementary table 1. The PCR primers list

| Genes | Primer sequences |
| --- | --- |
| *IL1A* | F: 5’- GGTTGAGTTTAAGCCAATCCA -3’  R: 5’- TGCTGACCTAGGCTTGATGA -3’ |
| *IL1B* | F: 5’- TACCTGTCCTGCGTGTTGAA -3’  R: 5’- TCTTTGGGTAATTTTTGGGATCT -3’ |
| *IL6* | F: 5’- CAGGAGCCCAGCTATGAACT -3’  R: 5’- GAAGGCAGCAGGCAACAC -3’ |
| *CXCL1* | F: 5’- CATCGAAAAGATGCTGAACAGT -3’  R: 5’- ATAAGGGCAGGGCCTCCT -3’ |
| *CXCL10* | F: 5’- GAAAGCAGTTAGCAAGGAAAGGT -3’  R: 5’- GACATATACTCCATGTAGGGAAGTGA -3’ |
| *CCL2* | F: 5’- AGTCTCTGCCGCCCTTCT -3’  R: 5’- GTGACTGGGGCATTGATTG -3’ |
| *TGFB1* | F: 5’- GGCCAGATCCTGTCCAAGC -3’  R: 5’- GTGGGTTTCCACCATTAGCAC -3’ |
| *GAPDH* | F: 5’- GTTGCCATCAATGACCCCTTCATTGACC -3’  R: 5’- CAGCATCGCCCCACTTGATTTTGG -3’ |

**SUPPLEMENTARY FIGURES**

**Supplementary Figure 1.** Cell viability of MDA-MB-231 and 4T1 cells after treated with increasing concentrations of Nif (0-0.5mM) or Pal (0–0.5mM) for 3 days.


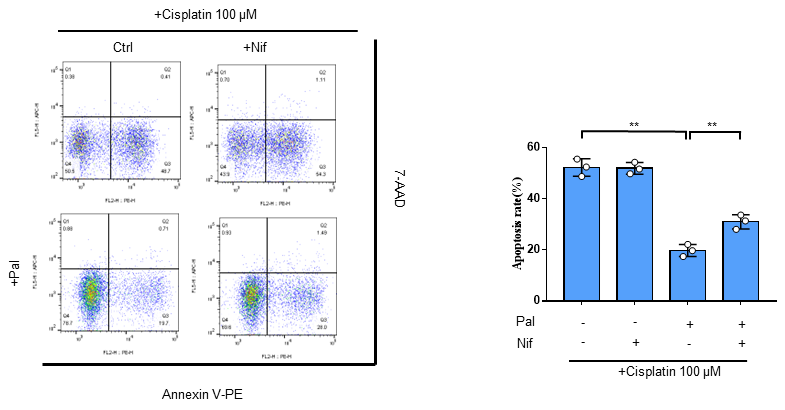


**Supplementary Figure 2.** After indicated treatment, medium was replaced and cells were following treated with cisplatin to induce apoptosis. Apoptosis rate was detected by flow cytometry through Annexin V/PI staining after 48h treatment.


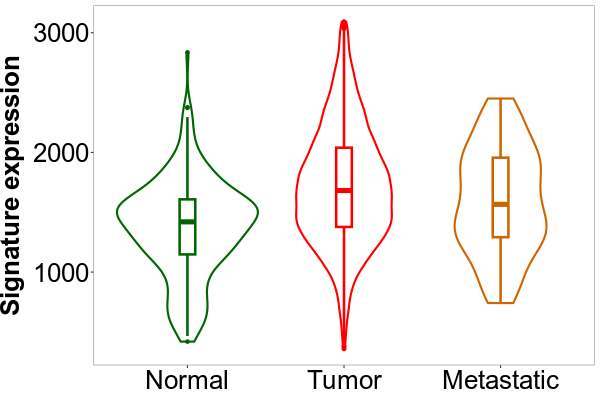

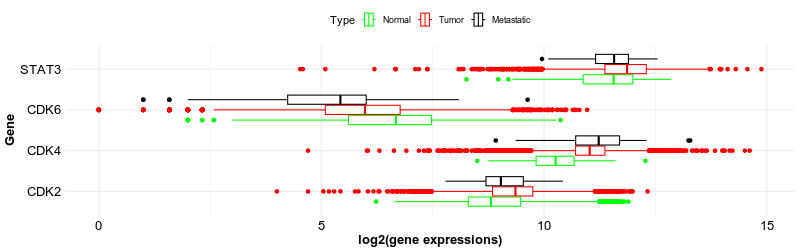


**Supplementary Figure 3.** The gene expression and gene signature of CDK2, STAT3, CDK4, and CDK6 were analyzed between normal breast tissue and breast tumors or metastatic breast tumors using the TNMplot database.
