# Supplementary material for: Nifuroxazide boosts the anticancer efficacy of palbociclib-induced senescence by dual inhibition of STAT3 and CDK2 in triple-negative breast cancer
Source: Cell Death Discov. 2023 Sep 26;9:355. doi: 10.1038/s41420-023-01658-w (PMC10522654; doi:10.1038/s41420-023-01658-w)
Supplement: Supplementary file 2 — Original Data File [file 41420_2023_1658_MOESM2_ESM.docx]

**Fig1 F**


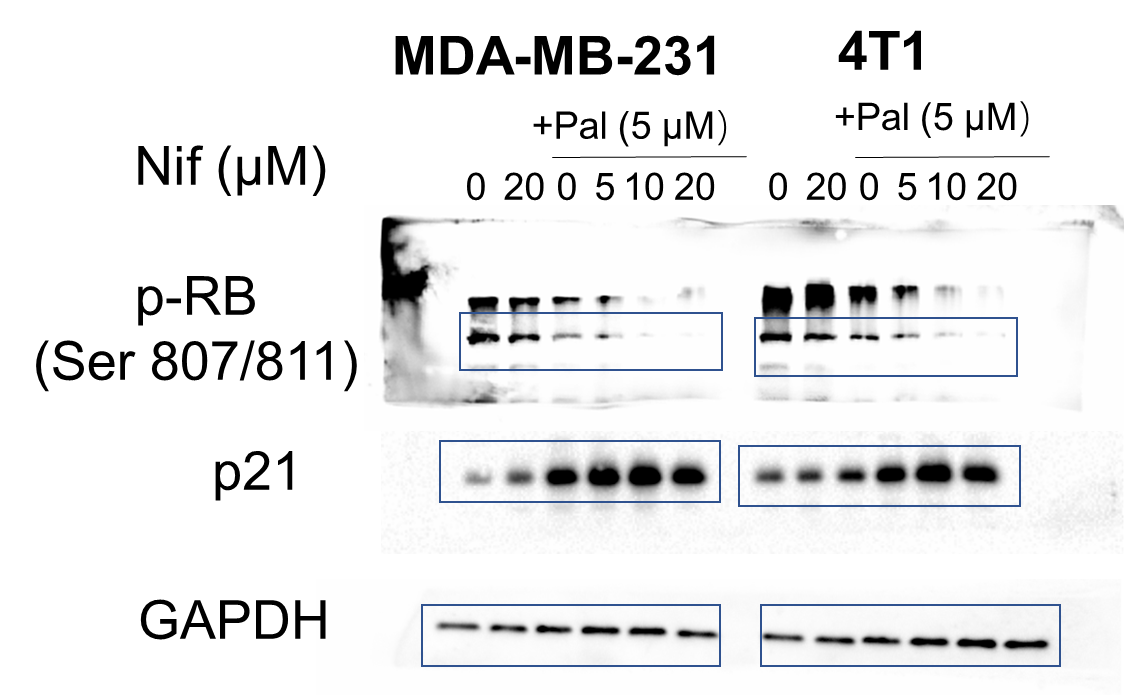


**Fig2 A**


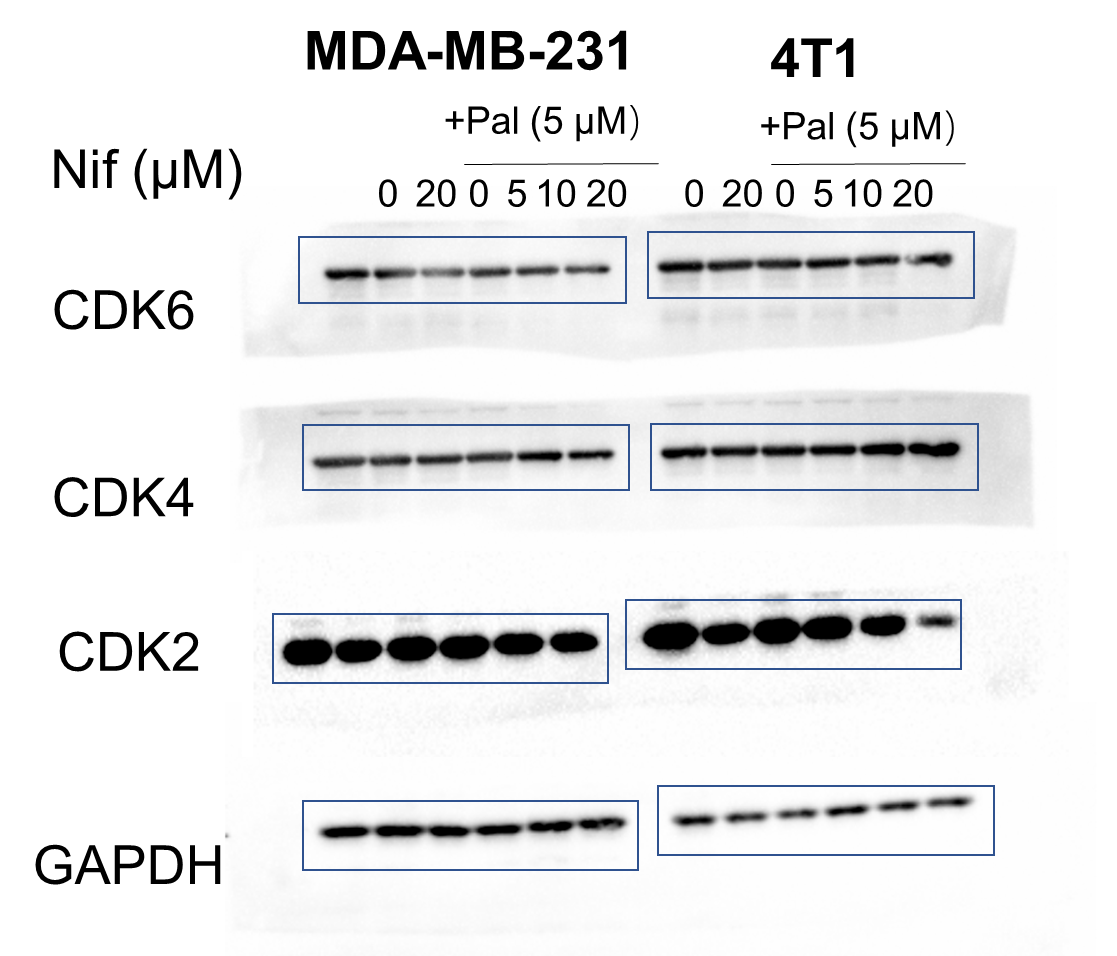


**Fig2 C**


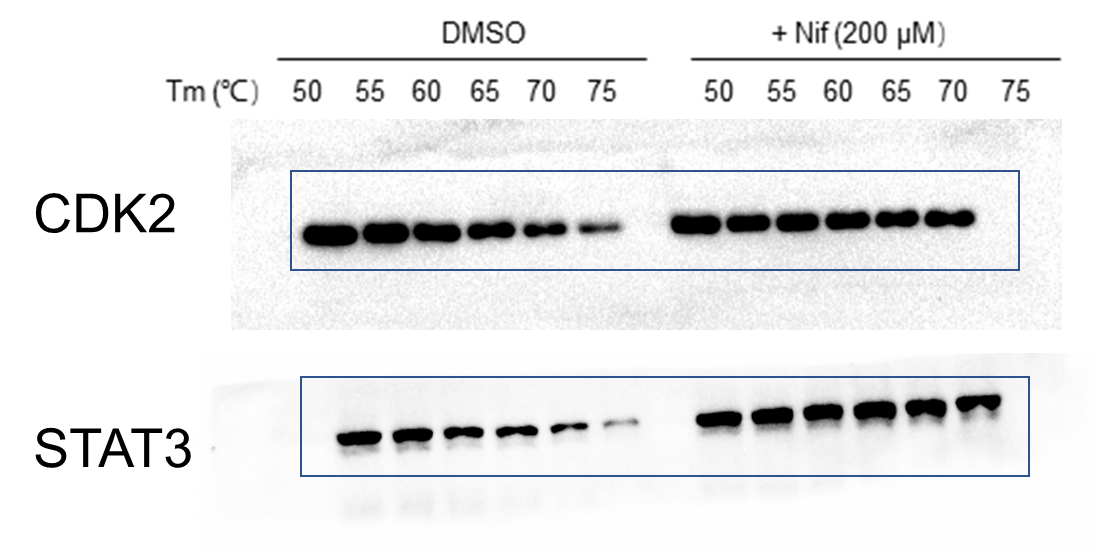


**Fig2 D**


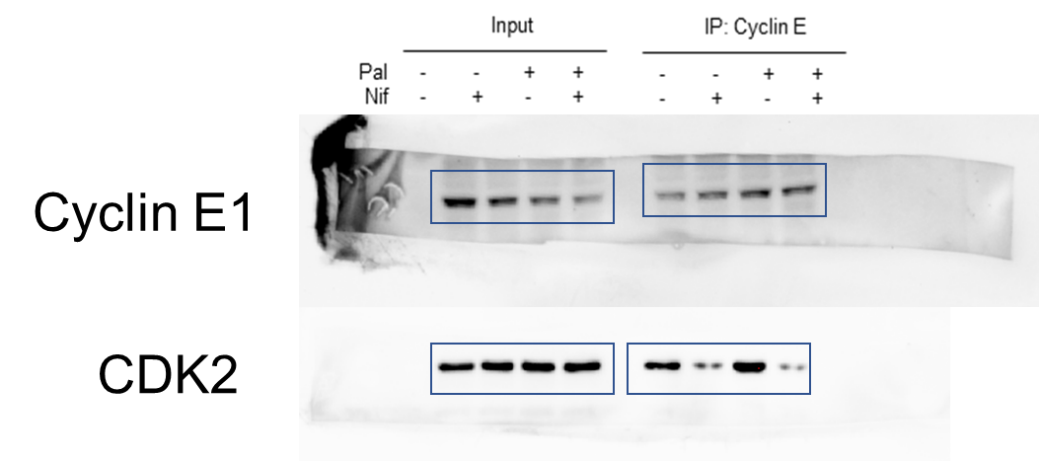


**Fig2 F**


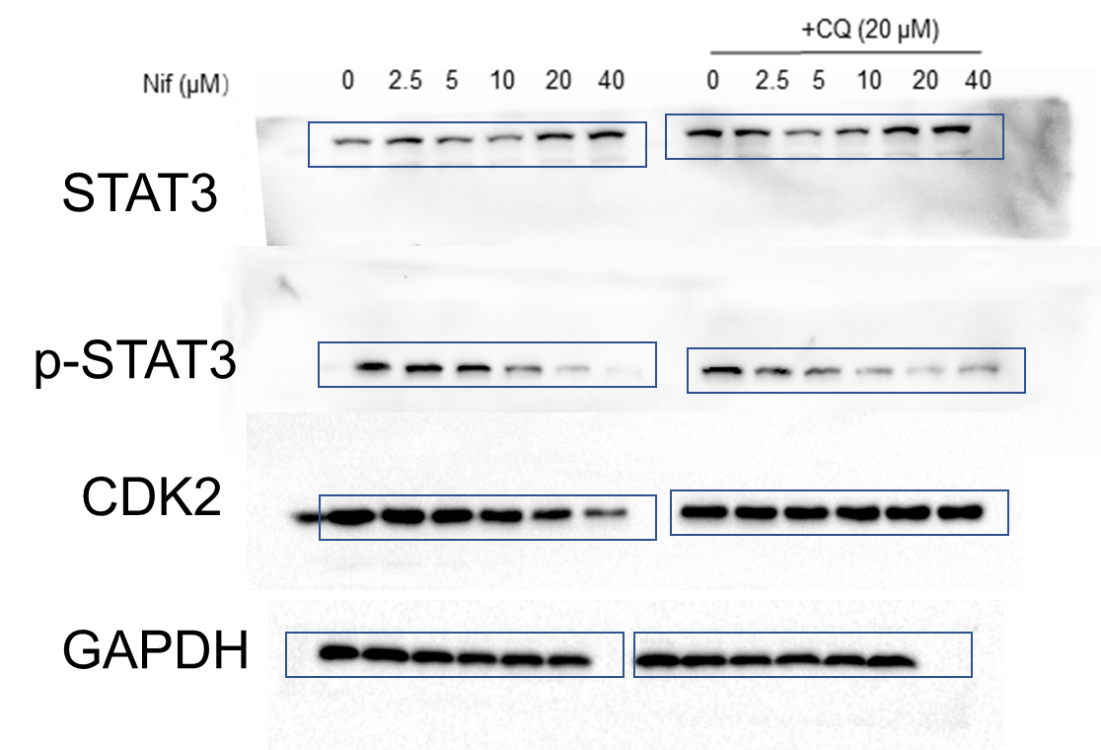


**Fig3 A**


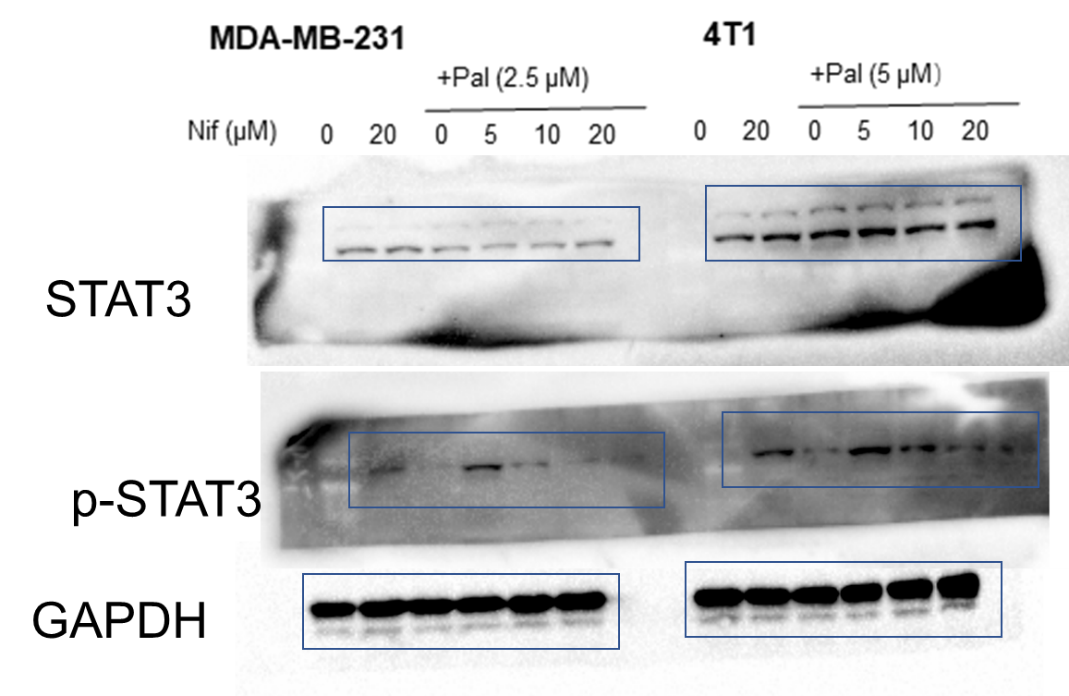


**Fig4 E**


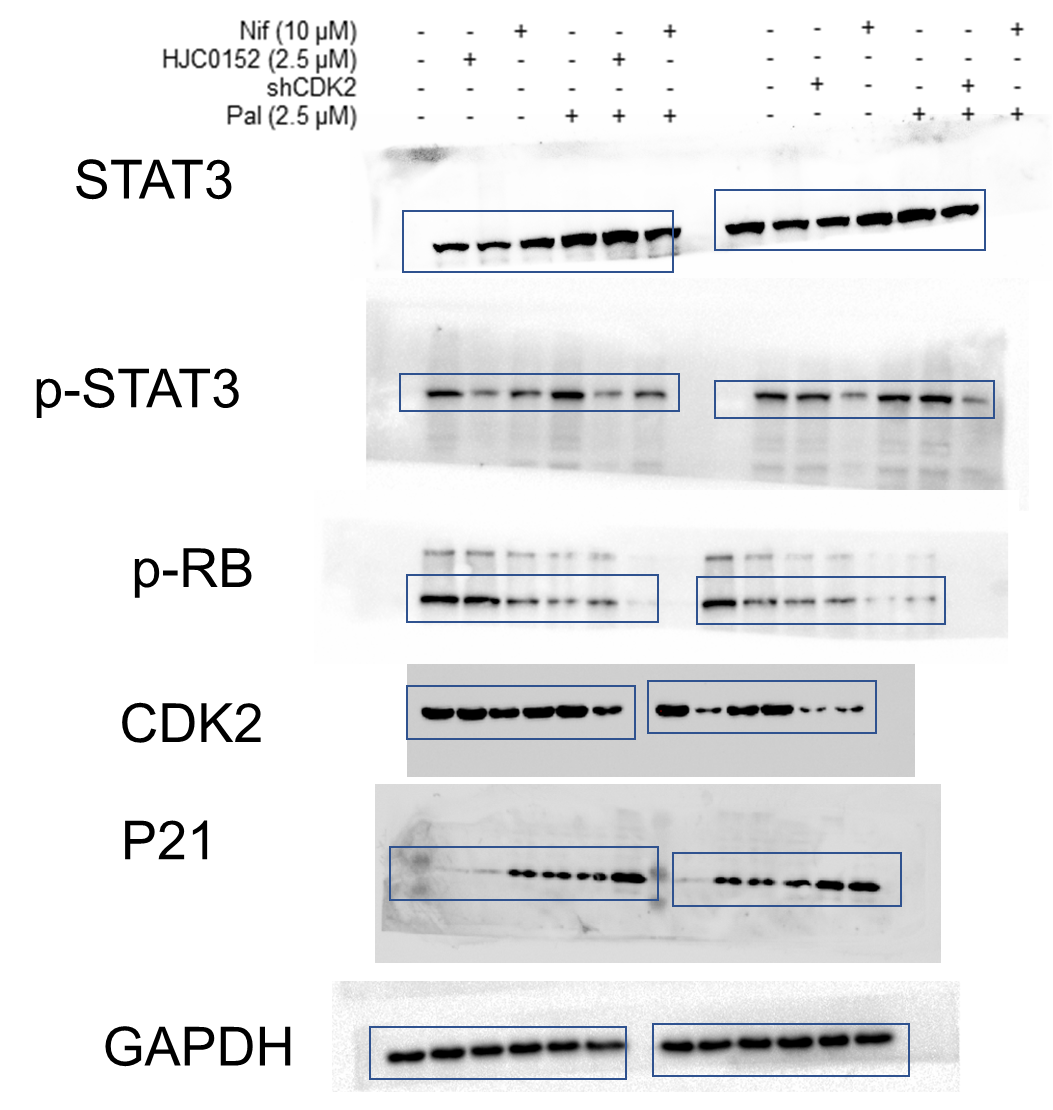


**Fig5 F**


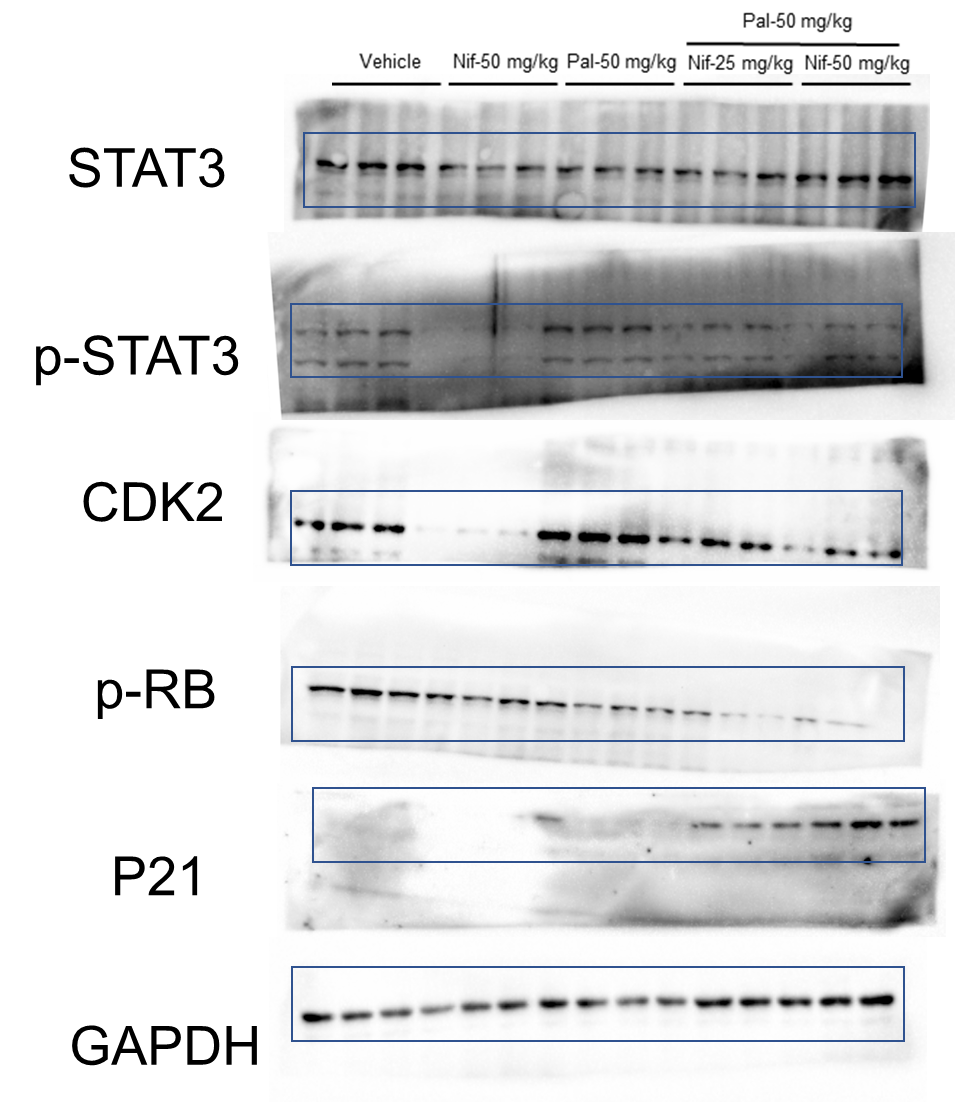


These are the images used for the final figures. All experiments were performed at least three

times and every protein was normalized with its own actin. The authors will provide the uncropped and full length images upon reasonable request
